# Investigating chromatin accessibility during development and differentiation by ATAC-sequencing to guide the identification of *cis*-regulatory elements

**DOI:** 10.1042/BST20210834

**Published:** 2022-05-23

**Authors:** Emily Louise Smith, Gi Fay Mok, Andrea Münsterberg

**Affiliations:** School of Biological Sciences, Cell and Developmental Biology, University of East Anglia, Norwich Research Park, Norwich, U.K.

**Keywords:** ATAC-seq, chromatin accessibility, *cis*-regulatory elements, enhancer, vertebrate development

## Abstract

Mapping accessible chromatin across time scales can give insights into its dynamic nature, for example during cellular differentiation and tissue or organism development. Analysis of such data can be utilised to identify functional *cis*-regulatory elements (CRE) and transcription factor binding sites and, when combined with transcriptomics, can reveal gene regulatory networks (GRNs) of expressed genes. Chromatin accessibility mapping is a powerful approach and can be performed using ATAC-sequencing (ATAC-seq), whereby Tn5 transposase inserts sequencing adaptors into genomic DNA to identify differentially accessible regions of chromatin in different cell populations. It requires low sample input and can be performed and analysed relatively quickly compared with other methods. The data generated from ATAC-seq, along with other genomic approaches, can help uncover chromatin packaging and potential *cis*-regulatory elements that may be responsible for gene expression. Here, we describe the ATAC-seq approach and give examples from mainly vertebrate embryonic development, where such datasets have identified the highly dynamic nature of chromatin, with differing landscapes between cellular precursors for different lineages.

## Introduction

Genomic DNA is packaged into chromatin composed of repeating structural units called nucleosomes, where 147 bps of DNA wrap 1.6 times around an octamer of core histone proteins. The degree of nucleosome occupancy determines chromatin accessibility, which is linked to biological functions, including gene expression, since physical access to regions such as gene promoters, enhancers or silencers is essential for transcription factor (TF) binding. Chromatin accessibility is facilitated by pioneer transcription factors, which bind to nucleosomes and recruit chromatin remodelling complexes, as well as other enzymes which modify histones and control DNA methylation to release DNA from the tightly coiled nucleosome into euchromatin, reviewed in [1–3].

During embryonic development and cell differentiation, the epigenetic and therefore chromatin landscapes change in response to developmental cues and external stimuli. Changes in access to chromatin can influence the activity of regulatory elements such as enhancers, short DNA sequences containing TF binding motifs that increase the likelihood of transcription of one or more genes through a *cis*-regulatory mechanism, whereby enhancer bound proteins assemble the transcription machinery to initiate transcription.

Enhancer identification remains challenging for several reasons. Enhancers can interact with promoters that are long distances away [4,5]. Although genome organisation imposes constraints on enhancer–promoter interactions [6] it is difficult to allocate gene-specific enhancers that are scattered throughout the genome. Models for interactions of enhancers with their target promoters include enhancer tracking, looping and loop-extrusion [7]. Experiments using the globin locus suggested that ‘looping out’ intervening DNA brings enhancer and promoter regions into proximity. Subsequent chromosome conformation capture experiments, which involve digestion and ligation of chromatin within formaldehyde cross-linked cells, confirmed this idea [8,9]. Furthermore, DNA sequence alone is not sufficient to identify enhancers, as sequence conservation is often limited to specific TF binding motifs, however some epigenetic marks are associated with enhancers. Finally, if the activity of an enhancer is spatially and temporally restricted, it cannot be identified unless the method used captures the correct tissue or developmental time point.

Functional characterisation of enhancers becomes difficult when several enhancers act on one gene to produce complex expression patterns. These can result from the cooperation between different enhancers with cell type or tissue-specific activities [10]. The presence of multiple enhancers with overlapping activity can provide robustness to gene expression and establish precise expression boundaries [11,12]. For example, in mouse embryos the Sonic Hedgehog (*Shh*) gene is controlled by two enhancers. When activated by both, *Shh* expression is restricted to the floor plate, but when controlled by only one enhancer, *Shh* transcription is confined to the limb bud [13].

Despite these challenges, many attempts have been made to identify enhancer elements, as this is critical in aiding our understanding of gene regulation, not only during embryonic development but also in human disease. Here, we review the use of ATAC-seq to aid enhancer discovery in dynamic systems.

## Overview of the ATAC-seq method

For enhancers to act on target promoters, chromatin must be accessible to enable TF binding, thus changes in chromatin accessibility usually correlate with enhancer activity. Therefore, chromatin accessibility assays have been used to identify *cis*-regulatory elements and active chromatin regions. Micrococcal nuclease sequencing (MNase-seq) is an indirect assay in which the enzyme digests any unprotected DNA. Nucleosomes can be purified and the bound DNA sequenced, generating an occupancy map of nucleosomes. Where there is a lack of nucleosomes, it is inferred that chromatin was more accessible. A direct method of chromatin accessibility includes formaldehyde-assisted isolation of regulatory elements (FAIRE-seq), which crosslinks DNA–protein complexes. Subsequent shearing by sonication, and phenol–chloroform extraction isolates nucleosome free regions, which can then be sequenced. Deoxyribonuclease I sequencing (Dnase-seq) and assay for transposase accessible chromatin sequencing (ATAC-seq) methods both work by utilising enzymes that recognise and cleave nucleosome depleted regions. For comparison of these methods, see [14].

Compared with these assays, which require a large starting cell count and are time consuming (∼50 million cells, 2–4 days), ATAC-seq [15] takes less than a day and works well with ultra-low input material, as few as 500 cells for a mini-bulk sequencing experiment. The simplicity of the protocol improves success rates and reproducibility of experiments and decrease probability of errors. The use of paired-end sequencing adaptors makes alignments of reads more accurate, for example mapping against repetitive regions of the genome.

**Figure 1.**
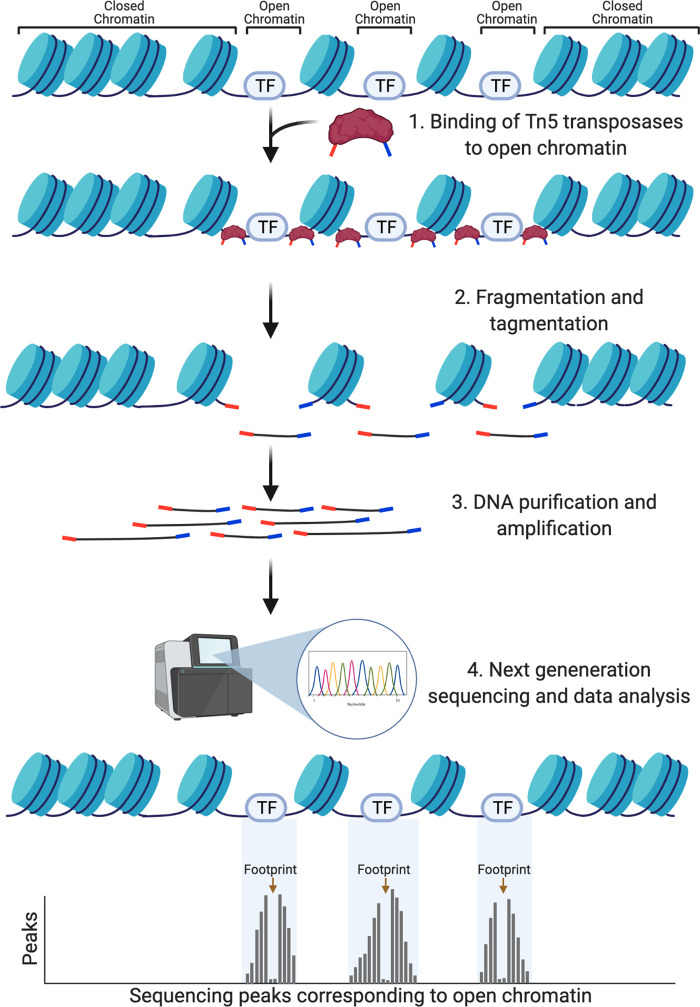
Identification of accessible chromatin regions using ATAC-seq. (**1**) Tn5 transposase (brown) inserts sequencing adaptors (red and blue) into regions of open chromatin. Nucleosomes are shown in light blue, DNA-bound transcription factors (TF) are shown in grey. (**2**) The chromatin is fragmented and simultaneously tagmented with sequencing adaptors. (**3**) DNA is purified and PCR-amplified into a library using barcoded primers. (**4**) The library can then be analysed by qPCR or next-generation sequencing (NGS). Data analysis is performed and accessible regions of chromatin show as peaks. Within peaks, lower read coverage indicates TF footprints and allows prediction of TF binding *in silico*.

In brief, the ATAC-seq protocol utilises a hyperactive prokaryotic transposase 5 (Tn5) enzyme to cleave open regions of chromatin and insert sequencing adaptors, a process known as tagmentation (Figure 1). Samples do not require fixation and the native chromatin state is analysed. DNA fragments are purified, amplified into a library using barcoded primers and directly sequenced using next-generation sequencing [15]. Sequencing reads are visualised in a genome browser and read number positively correlates with accessibility of that region of chromatin. With sufficient sequencing depth, footprints of bound TFs are also detectable *in silico*, within accessible peaks; see for example [16]. The protocol is useful when sample cell populations are small, rare, or difficult to obtain, such as specific cell populations within an embryo. Indeed, ATAC-seq can be performed on single cells [17] (see below). Limitations of ATAC-seq include the potential contamination of data with mitochondrial DNA and potential sequence or structural biases of the Tn5 enzyme [18,19]. Overall however, the speed, scalability and ease of experiment makes ATAC-seq a popular approach for studying chromatin in dynamic systems.

## Development of single cell ATAC

Whilst bulk ATAC-seq has many advantages, it cannot determine chromatin accessibility of individual cells within a heterogenous population. To overcome this issue, ATAC-seq has been adapted for single cells (scATAC-seq), with two protocols published at the same time. One relies on the physical isolation of individual cells by microfluidic chambers (C1, Fluidigm), followed by tagmentation, library generation and sequencing [17]. Alternatively, combinatorial barcoded primers mark individual cells. Pools of isolated nuclei undergo tagmentation with unique barcoding primers in 96 well plates. This is followed by re-pooling, sorting into new 96-well plates, to which a second round of barcoded primers are added during PCR amplification. Each of these pools are sequenced, and there is a low probability that cells within the same pool share the same combination of barcodes [20,21]. Both methods have relatively low throughput and provide lower complexity data, however, recent optimisations resulted in increased cell number, enhanced recovery of nuclear fragments per cell and improved data quality [21–23].

As it is technically more demanding, fewer scATAC-seq experiments have been performed. In addition, minor sub-populations can be hard to detect and the bioinformatic analysis of scATAC-seq is more time consuming [24] — for a review of analytical procedures see [25,26]. An excellent alternative is mini-bulk ATAC-seq, for example on FACS-isolated cells or on affinity purified nuclei, which can provide novel insights into discreet sub-populations of cells, such as avian neural crest cells [27,28], zebrafish endothelial cells [29] or Drosophila blastoderm cells [30].

Despite quickly and accurately identifying regions of open chromatin, ATAC-seq cannot on its own identify the type of regulatory element, such as an enhancer, silencer, or promoter region. However, coupled with other genomic techniques ATAC-seq is very useful to identify active regulatory elements in different cell types.

## Using ATAC-seq with other genome-wide approaches

Epigenetic marks that characterise enhancer regions include various histone modifications. Mapping these features by ChIP-seq, or Cut and tag techniques [31], can determine genome wide protein–DNA interactions. Histone acetylation is generally associated with active chromatin, whereas mono-methylation of histone H3 at lysine 4 (H3K4me1) marks inactive regions. Acetylation can destabilise nucleosome-DNA interactions. In particular, acetylation of histone H3 at lysine 27 (H3K27ac) by P300 and CREB marks active enhancers. ChIP-seq datasets for P300, CREB and histone marks accurately identified novel enhancers in mouse [32,33]. Additional post-translational modifications are increasingly observed for different enhancer states and other histone marks are associated with subsets of enhancers [34]. While future work is required for complete annotation of all histone modifications that are indicative of CREs, correlating independent ATAC-seq and ChIP-seq datasets can increase the likelihood of identifying active enhancer regions [5].

Furthermore, multi-omic experiments can combine ATAC-seq with other genomic techniques in the same sample. This is advantageous when working from limited starting material. For example, combining RNA-seq with ATAC-seq enables simultaneous profiling of chromatin dynamics and changes in gene expression patterns. Sci-CAR uses a pooled barcode method [35], other methods that do a similar comparison have also been produced [36,37]. ATAC-ME is an integrated method to investigate accessibility or TF binding and DNA methylation from a single DNA preparation [38]. EpiMethylTag also combines ATAC-seq or ChIP-seq with bisulfite conversion to examine accessibility and methylation patterns in the same sample [39,40].

ATAC-seq is a powerful tool in assessing the chromatin landscape of different cell and tissue types. When used in combination with other genomic approaches, it can provide insight into the epigenome of specific cell populations. Since its introduction, ATAC-seq has been optimised for different purposes. For example, the Omni-ATAC protocol has been adapted for use with a wide variety of tissue types, including snap-frozen tissues, and yields a higher number of peaks and reduced mitochondrial reads [14,41].

## Dynamic patterning processes in developing embryos

Investigating the accessible chromatin landscape is of particular interest when looking at dynamic changes. For example, during the complex process of embryo development gene expression patterns are changing rapidly and are associated with changes in cell states during cell fate acquisition and differentiation programmes. With ATAC-seq it is possible to generate sufficient replicates for comparisons between different samples, such as developmental time points or tissues. This facilitates atlas-projects for multiple tissues, as for example in mouse [42] and zebrafish [43]. The differential analysis of chromatin accessibility can highlight regions of interest for validation and functional testing. The following sections give selected examples where ATAC-seq has provided insights into chromatin accessibility during normal development, tissue differentiation and regeneration, or in mutant or disease scenarios. These are summarised in Table 1.

**Table 1:** Studies using ATAC-seq in model organisms for development

Species	Biological context	Reference
Drosophila	Domain-restricted analysis for anterior–posterior patterning of blastoderm to identify accessible regions	[30]
	Tissue-specific accessibility during three embryonic stages with germ-layer enhancer validation	[44]
Zebrafish	Chromatin accessibility atlas of embryonic and adult tissues	[43]
	Identification of key elements during zygotic genome activation	[45]
	Neural crest and melanoma development	[53,56]
	Heart regeneration	[64,67]
	Liver development and response to injury	[68]
	Fin regeneration	[69]
	Endothelial enhancers	[29]
Xenopus	Wnt signalling in dorsal–ventral patterning in comparison with mesoderm and neural crest development	[47]
	Mesendoderm specification	[48]
Chicken	Neural crest development, GRN reconstruction and identification of specific enhancers	[27,28]
	Anterior–posterior axis elongation and paraxial mesoderm development, differential TF occupancy and *in vivo* enhancer validation	[16]
Mouse	Chromatin accessibility atlas of adult tissues	[42]
	Sex-specific accessibility of *in vivo* and IVF inner cell mass	[50]
	Chromatin accessibility preconfigures region-specific neural fates along anterior–posterior axis	[52]
	Sinoatrial node development	[65]
	Heart development of key developmental stages	[66]
Mouse/Pig	Limb development and digit adaptation	[63]
Bovine	Chromatin accessibility in oocytes and early embryos, and comparison of *in vivo* and *in vitro* blastocysts	[49]
Human	Chromatin accessibility of inner cell mass and trophectoderm of blastocysts	[51]
	Human ESC differentiation into neural crest identifies disease enhancer	[5]

## Early events: gastrulation, germ layer formation and axis patterning

In Drosophila embryos, gene-regulatory networks (GRN) that pattern the anterior–posterior axis are well characterised and involve gap and pair-rule genes. ATAC-seq was performed on cellular blastoderm-stage embryos, shortly after zygotic genome activation. Seven well characterised enhancers were used to tag blastoderm nuclei for affinity purification. The regional variation in chromatin accessibility observed, correlated with regulatory activity of axis patterning enhancers, suggesting mechanisms by which transcriptional activator and repressor proteins modulate enhancer accessibility [30].

Chromatin accessibility dynamics have also been investigated using a developmental series of Drosophila embryos. Three major embryonic stages were examined using scATAC [44]. Interestingly, differential chromatin accessibility revealed that cellular heterogeneity was already present in the blastoderm, and individual cell types could be inferred before the major lineages are specified during gastrulation. Among a large number of candidate regulatory elements with tissue-specific accessibility (>30 K) a subset was validated in transgenic embryos. Importantly, the germ-layer specific activity of these predicted enhancers was accurate in 90% of cases, demonstrating the power of this approach.

ATAC-seq during early zebrafish development, from zygotic genome activation to the onset of lineage specification, showed that chromatin accessibility increased at regulatory elements, often preceding transcription of the associated genes [45]. Loss of maternal TFs (Pou5f3, Sox19b, Nanog) led to decreased accessibility suggesting that they open up chromatin during genome activation.

An important transition, discovered in Xenopus, is the loss of competence to respond to dorsalising Wnt signals in late blastula stages [46]. This is associated with reduced accessibility at Wnt-responsive promoters [47]. Integration of ChIP-seq, ATAC-seq and transcriptomics data using machine learning uncovered novel TFs involved in mesendoderm formation during Xenopus gastrulation [48].

In mammalian systems, chromatin accessibility also increases during genome activation in bovine embryos [49]. In both bovine and mouse blastocysts, differences in accessibility patterns were identified between those generated *in vitro* and *in vivo*, providing insights into features that could be important for successful preimplantation development [49,50]. The differential accessibility in mouse correlated with differential expression of genes related to stress signalling and cardiac hypertrophy signalling, consistent with the idea that effects on health could originate at preimplantation stages with exposure to environmental stress [50]. Finally, differences in chromatin accessibility have been characterised in human embryos between embryonic inner cell mass and extra-embryonic trophectoderm tissues [51].

## Epigenome regulation of ectoderm-derived tissues

Several studies have examined chromatin accessibility during vertebrate neural and neural crest differentiation using embryonic stem cells, zebrafish, mouse and chick embryos [27,28,52–54]. ATAC-seq identified differentially accessible regions in mouse embryonic stem cells (ESC) differentiated into neural progenitors with different anterior–posterior identities. These regions were similar to those present *in vivo*, in hindbrain or spinal cord progenitors suggesting that chromatin accessibility preconfigures region-specific neural fates [52].

The dorsal-most aspect of the neural tube generates the neural crest (NC), a vertebrate-specific population of cells. Different NC sub-populations arise along the axis, characterised by distinct migratory patterns and developmental potential, generating diverse cell types, including craniofacial bones, septa of the heart, neurons and glia of the peripheral nervous system, and pigment cells of the skin. Multiple genomics approaches, including ATAC-seq and RNA-seq, have elucidated the epigenomic mechanisms that govern (NC) formation and establish their axial level identity (see reviews by [54,55]). Work in chick embryos used FACS to isolate cranial NC cells at different stages of migration [27], as well as vagal NC cells which generate the enteric nervous system [28]. Chromatin and transcriptional landscapes were characterised at different time points, this identified regions of differential chromatin accessibility and allowed reconstruction of NC-specific GRNs. Both studies validated several NC specific enhancers *in vivo*, by chick embryo electroporation. The differential activity of these enhancers uncovered heterogeneity at the regulatory level and distinct vagal NC populations already predetermined prior to neural tube delamination.

The same group used genetic labelling in zebrafish combined with FACS isolation of NC populations from wildtype or FoxD3 mutants. These were subjected to RNA-seq, ATAC-seq and H3K27ac ChIP-seq. Combining *in vivo* biotinylation of FoxD3 with ChIP-seq showed that FoxD3 initially acts as a pioneer factor for NC specification genes, before switching to a transcriptional repressor function [53].

The importance of enhancer discovery and functional characterisation is illustrated by a study looking at the congenital craniofacial disorder, Pierre Robin Syndrome (PRS) [5]. *In vitro* differentiation of human ESC into cranial NC cells combined with detection of epigenome marks highlighted differentially accessible ATAC-peaks that were overlapping with p300 and H3K27ac/H3K4me histone marks. This identified three putative NC specific enhancers located 1.45 Mbp distant to the chondrogenic TF, Sox9, in a region that is deleted in PRS patients. Transgenic LacZ-reporters confirmed enhancer activity in mouse craniofacial development, and ATAC-seq of hESC-derived cranial NC cells confirmed accessibility during a restricted window of development prior to chondrogenic differentiation.

Similarly, ATAC-Seq analysis of multiple zebrafish melanoma tumours identified a developmental enhancer that is also important in cancer [56]. Accessible peaks were validated using EGFP reporter constructs in transgenic zebrafish. This demonstrated activity *in vivo*, of a region 23 kb upstream of Sox10, a key TF for NC development that is also up-regulated in melanoma initiation.

## Chromatin accessibility in paraxial mesoderm and limb buds

As highlighted in the examples above, ESC differentiation protocols and transgenic reporter systems in Drosophila, zebrafish and mouse provide excellent assays for validation of candidate enhancers and their functional analysis. The avian system is also very useful for this purpose due to the ease with which embryos can be obtained and manipulated. Following identification of differentially accessible candidate regulatory elements, electroporation of enhancer-reporter plasmids [57,58] combined with live imaging [16] allows the rapid analysis of spatio-temporal enhancer activity *in vivo*. Mutagenesis of TF binding sites in enhancer-reporter plasmids, followed by epigenome editing of endogenous enhancer elements confirms their functional importance [16,59].

We recently characterised the open chromatin landscape in vertebrate paraxial mesoderm in the chicken embryo [16]. As the body axis extends, the mesoderm on either side of the neural tube produces paired segments, termed somites. Developmental signals control somite differentiation and the emergence of cell lineages of the musculoskeletal system, such as chondrocytes and skeletal muscle cells. This process generates a spatiotemporal gradient of differentiation, by using a combination of ATAC-seq and RNA-seq we identified changes in gene expression signatures and accessible chromatin that occur in paraxial mesoderm along the axis. Differentially accessible chromatin regions within HOX clusters were associated with axial identities and *in silico* footprint analysis revealed the differential coverage for a number of TFs, known to be involved in axis patterning or cell differentiation, such as CDX2, HOX paralogs, PAX3, TWIST2 or LEF1. Correlation of accessible chromatin with expressed genes helped to identify candidate regulatory elements, which were validated *in vivo*. Electroporation of fluorescent enhancer reporters into early chick embryos demonstrated the restricted activity of enhancers located upstream of TCF15 and MEOX1 genes. Time-lapse imaging could detect the onset of enhancer activation *in vivo* and the importance of candidate TF motifs was confirmed by mutation analysis. CRISPR-mediated epigenome editing led to loss of gene expression and phenotypic changes confirming the importance of the enhancers for vertebrate axis development [16].

Morphological changes in the skeletal elements of tetrapod limbs are a fascinating model for evolutionary adaptations, and GRNs underlying limb outgrowth and development have been studied extensively in chick and mice [60,61]. The polarised expression of Shh in the posterior limb bud mesenchyme is particularly important for digit patterning [62]. A recent study examined the molecular mechanisms leading to digit adaptations in pigs versus mice [63]. Interspecies comparison of ATAC-seq data was used to examine regulatory changes associated with the morphological changes in these two species. Whilst many accessible chromatin regions were conserved, there were some divergent regions associated with genes encoding components of signalling pathways required for limb development. This is intriguing, however, the functional relevance of these elements for species-specific digit patterning remains to be confirmed.

## Epigenome regulation in the heart, endothelial cells and during regeneration

The heart and associated vessels are crucial for survival, and several studies have examined epigenetic changes driving cell fate transitions during cardiovascular development and regeneration [29,64–67]. Cardiac progenitor cells (CPC) marked by Nkx2–5 and Isl1 expression were examined in early mouse embryos, between E7.5 to E9.5 [66]. Combining single-cell RNA sequencing with bulk- and scATAC-seq allowed better characterisation of GRNs that govern cell fate transitions and identified cardiac sub-populations and their developmental trajectories. CPCs expressing Nkx2–5 committed to a cardiomyocyte fate and open chromatin states, which depend on both Isl1 and Nkx2–5, were associated with CPC fate transitions.

Postnatally, Isl1 is also expressed in cardiac pacemaker cells (PC) a specialised type of cardiomyocyte located in the sinoatrial node (SAN) and critical to initiate the heartbeat. Comparative ATAC-seq was used to investigate accessible chromatin that governs PC-specific gene expression [65]. FACS of dissected neonatal mouse SAN was used to isolate PCs, which were compared with right atrial cardiomyocytes. This identified differentially accessible peaks and SAN-specific enhancers, which were confirmed by transgenic enhancer LacZ-reporter mice.

In the mouse, cardiac regenerative capacity is limited to neonatal stages. However, in adult zebrafish several tissues can regenerate, and this has been used to determine chromatin accessibility, including in the heart following cryoinjury or ventricular resection injury [64,67], or the liver [68], or tail fin [69]. In regenerating cardiomyocytes, chromatin accessibility changes extensively [64], this may be regulated by the AP-1 TFs, Junb and Fosl1, found to be enriched in ATAC-seq peaks. Thus, the AP-1 TFs may promote the regenerative process by activating gene expression programmes important for cardiomyocyte dedifferentiation, proliferation and migration. Additional ATAC-peak-enriched motifs were identified in epicardium after ventricular resection [67], including TFs known to regulate epicardium development and/or regeneration, such as Tcf21, Runx1, TEAD, C/EBPb, Smad2/3/4 and Gli2. This study also highlighted TFs not previously linked to epicardial functions. Several candidate enhancer regions correlated with enriched H3K27ac marks and nearby genes showed increased gene expression in epicardial cells after injury. In addition, transgenic EGFP-reporters confirmed injury-induced activity for these regulatory elements. Enhancers important for zebrafish fin regeneration were also recently identified by comparing ATAC-seq data from uninjured and regenerating caudal fins [69]. This identified regions of DNA with dynamic accessibility during regeneration. The differential peaks were assigned to nearby genes through association with corresponding gene expression changes detected by RNA-seq. Experiments in transgenic zebrafish validated several novel regulatory sequences near fin regeneration genes.

Transgenesis in zebrafish is a versatile approach and fluorescently labelled endothelial cells (fli1a:egfp) were recently used to discover active enhancers [29]. ATAC-seq was performed on sorted nuclei, and fluorescent and non-fluorescent populations were compared. This identified more than 5000 elements that were differentially accessible in endothelial cells and located in the vicinity of genes known to be important for vascular development. Functional experiments in zebrafish embryos then validated enhancers controlling endothelial-specific gene expression.

## Conclusion

Here we highlighted the ease with which chromatin accessibility can be characterised using ATAC-seq. Combination of ATAC-seq with other genome wide approaches, in particular transcriptomics and epigenome modifications, enhances its power. The chosen examples illustrate detection of differential chromatin accessibility in a number of dynamic biological systems. We emphasise the importance to validate candidate regions of interest, which is crucial to confirm their functional relevance, but is inevitably more involved when using *in vivo* models.

## Perspectives

- The genome-wide capture and analysis of chromatin accessibility by ATAC-seq is highly feasible across many species and tissue types. Differential analyses identify regions with restricted spatio-temporal accessibility. Contingent on sufficient sequencing depth, ATAC-seq can elucidate TF footprints *in silico* and thus provide information for further functional testing and validation.
- ATAC-seq in combination with transcriptomics and computational analyses can indicate epigenome-transcriptome interactions to elucidate gene regulatory networks (GRN). Isolation of cells by tagging or FACS limits heterogeneity and helps to define discreet sub-populations for analysis.
- The combined use of multiple genomics techniques, such as ATAC-seq for chromatin accessibility, ChIP-seq for epigenome marks and RNA-seq for gene expression profiling, is a powerful approach to study the regulation of the epigenome during dynamic processes. The approaches can be applied to study normal embryo development or in scenarios where there is an experimental treatment, genetic mutation or disease model.

## Abbreviations

ATAC-seq: ATAC-sequencing
CPC: cardiac progenitor cells
ESC: embryonic stem cells
GRN: gene-regulatory networks
NC: neural crest
PC: pacemaker cells
PRS: Pierre Robin Syndrome
SAN: sinoatrial node
TF: transcription factor
